# Developing an AI framework for learning in higher education: a humanities perspective from English Literature

**DOI:** 10.1186/s41239-025-00562-w

**Published:** 2025-11-03

**Authors:** Bridgette Wessels

**Affiliations:** Sociology, School of Social and Political Sciences, University of Glasgow, 42 Bute Gardens, Glasgow, G12 8RT UK

**Keywords:** AI, Active learning, Chaucer studies, Learning and teaching, Higher education, ChatGPT, Amazon Polly

## Abstract

Little is known about how to develop frameworks for using AI to support learning in English Literature within higher education. This paper presents research that developed a framework for employing AI in the study of the fourteenth-century English poet Geoffrey Chaucer. Studying Chaucer presents challenges for modern readers, as it requires an understanding of his literary style, spoken Middle English, and the cultural and social context of the late medieval period. The research investigated how AI tools, such as ChatGPT and Amazon Polly, could support the study of Chaucer. It found that while AI can assist with students’ initial familiarisation with Chaucer’s poetry, it lacks the depth and rigour necessary for more advanced study. As a result, students and lecturers still rely on traditional resources, including authoritative print-based and digital scholarly editions. Consequently, the research developed a framework for studying Chaucer's poetry that integrates the guided use of AI through active learning with additional supporting resources, such as digital scholarly editions.

## Introduction

Little is known about how to develop frameworks for using AI in student-centred learning in English Literature within Higher Education (HE). This paper discusses research that developed a framework for applying AI to the study of the fourteenth-century English poet Geoffrey Chaucer. Chaucer is traditionally referred to as the “Father of English Poetry” and is best known for The Canterbury Tales, a collection of fourteen stories narrated by a group of pilgrims traveling from London to Canterbury Cathedral. Studying The Canterbury Tales involves understanding the English language of the fourteenth century (referred to as Middle English), Chaucer's literary style, and the culture and society of late medieval England, as revealed through the conversations of his humorous and often morally ambiguous characters. Students typically use printed, digital, and oral resources when studying Chaucer, and they are now beginning to explore the use of Generative AI.

In general terms, the commercial mainstreaming of Generative AI tools such as ChatGPT and neural language processing services, such as the text-to-speech tool Amazon Polly, raises questions about how AI may feature in learning and teaching within Higher Education. Successfully using Generative AI requires an understanding of its benefits and limitations, as well as the type of learning and teaching framework it necessitates. UNESCO’s *AI and Education: Guidance for Policy-Makers* (Miao et al., 2021) and its 2024 *Competency Frameworks for Students and Teachers* advocate for a human-centred approach. This approach emphasises that AI should support human decision-making and intellectual development, which are integral to active learning. Despite growing interest, there remains little empirical research on developing AI for learning and teaching (Chan, 2023). This paper contributes to the field by presenting empirical findings on the use of AI in the study of Chaucer.

This paper is based on the project *C21 Editions: Scholarly Editing and Publishing in the Digital Age* (hereafter C21 Project). The aim of the project was to develop a learning and teaching framework for using AI in the study of Chaucer based on student and lecturer practices. The objectives were: (1) to explore how ChatGPT and Amazon Polly could be used in studying Chaucer, and (2) to assess the extent to which expert supervision and resources are needed to oversee and improve these approaches. The project integrated digital humanities, sociology, Chaucer studies, and participatory design approaches in an inductive and iterative research process. The research was conducted with students, lecturers, and Chaucer experts at two universities in the UK and the US.

This paper first introduces the context of AI in learning and teaching within HE, including active learning. Second, it outlines the methodology of the C21 Project. Third, the findings are presented, followed by a discussion in Section Four. Finally, the Conclusion summarises the paper's contributions, outlines areas for further research, and offers recommendations.

## Context: AI in learning and teaching within HE

There is growing recognition that AI, including Generative AI, may offer benefits for learning and teaching within HE (UNESCO, 2024). However, concerns remain regarding its use, including the depth and accuracy of its outputs, the quality of the data on which it is trained, the potential for undetected bias, and the risk of misuse; for instance, students using ChatGPT to write their essays (Chan, 2023). These issues not only undermine the integrity of academic standards but also hinder students’ development as active learners with the critical skills needed to assess AI use (Zhou & Schofield, 2024). Despite these concerns, Zhou and Schofield (2024) argue that AI has the potential to support learning at the HE level. Scholars such as Chan (2023), and organisations such as UNESCO (2024) and the Council of Europe (2024), argue that universities must develop frameworks to guide the use of AI in learning and teaching. These frameworks must consider both pedagogical practices and the various types of AI systems.

In HE, learning and teaching are shaped by the principles of active learning, broadly defined as where ‘students are engaged in the learning process as participants’ (Matinez & Gomez, 2025, p. 43). More specifically, it is defined as ‘instructional activities involving students in doing things and thinking about what they are doing’ (Bonwell & Eison, 1991, p.iii). Drawing on experiential learning and learning by doing (Dewey, 1938), active learning uses a variety of participatory strategies, including discussion, flipped classroom, experimental and problem-based learning, and use of digital resources (Zhao et al., 2020). These encourage students to actively construct understanding and knowledge, and to reflect on their learning process (Shah & Campus, 2021). The role of lecturers in active learning is facilitative, guiding students in their learning (Serin, 2018). It involves them aligning activities with learning objectives, supporting student interactions, incorporating self-reflection, debriefing to consolidate learning and gathering continuous feedback (Dzaiy & Abdullah, 2024).

Through active learning, students critically engage with texts, digital resources, and academic debates, and draw on their own experiences in the course of academic study (Rasul et al., 2023). When studying Chaucer, for example, students need to understand the content, rhyme, and metre of his poetry; the Middle English language; and the social, religious, and cultural context of medieval England. They are also encouraged by lecturers to reflect on how Chaucer’s work relates to contemporary issues. Students engage with a variety of texts and scholarly debates, and use a wide range of resources, including digital, oral, and printed materials. Through this, they develop skills in analysis, evaluation, and problem solving (Wang, Zhan & Li, 2022) in becoming independent and critical learners (Wathall, 2024). This extends into how students can learn to use AI in informed and reflective ways and how lecturers can guide them to use it in constructively. An important element of any AI framework is identifying which types of AI applications are most appropriate for specific learning purposes (Crompton & Burke, 2025). There are many AI tools available for personalised learning, teacher support, and accessibility. However, little is known about how Generative AI tools such as ChatGPT, and neural language applications such as Amazon Polly, can be used effectively in learning and teaching.

Equally important is the need to address the potential negative effects of AI. These include students relying on AI to write essays, summarise texts instead of reading them in full, or replace independent research with AI-generated content (Chan & Colloton, 2024).

To address these issues, Chan (2023) argues that empirical research is needed to understand how best to support students in using AI tools like ChatGPT effectively in their academic work. Students must be equipped to use AI responsibly and ethically, in ways that uphold academic integrity (Cotton et al., 2023). These competencies are essential not only for academic success but also for preparing students for the workplace. They also reflect the calls by UNESCO and the Council of Europe for the responsible and informed use of AI.

The use of AI in HE takes place within a broader context of expanding digital and data-driven resources. Research-based digital tools and datasets are now integral to the HE learning environment (Goldie, 2016). These include MOOCs, digital platforms such as Moodle, and specialised resources like the George Thomason Newsbooks, which allow users to annotate texts and generate insights into historical topics. Students also have access to official online datasets, user-generated content from social media, and other digital sources such as websites and conference papers. These resources complement traditional printed books and peer-reviewed articles, supporting and enhancing active learning.

## C21 project methodology

The C21 Project employed a qualitative methodology to understand the perspectives of students, lecturers, Chaucer experts, and digital humanities developers regarding the use of AI. It aimed to explore how students and lecturers were using AI in learning and teaching, as well as to understand Chaucer experts’ views on key issues in studying Chaucer. This approach was adopted to develop a framework based on the learning and teaching requirements of students and lecturers.

The research design was inductive, collaborative, and iterative. It began with the premise that using AI in Chaucer Studies requires the “artful integration” (Suchman, 2003) of learning and teaching practices with supporting resources. Artful integration involves collaboration among people with diverse insights in research-led design (Suchman, 2003). In the C21 Project, this meant involving students, lecturers, Chaucer experts, and digital humanities developers throughout the research process. To support the artful integration of participants’ perspectives, the project focused on the use of different AI systems for the learning and teaching of Chaucer, specifically ChatGPT for understanding Chaucer's poetry as a written text, and Amazon Polly for understanding it as a spoken text. The project used *The Pardoner’s Prologue & Tale*, one of the tales from *The Canterbury Tales*, as a source to test AI tools and design any required supporting resources.

The project first explored Chaucer experts’ perspectives on resources for studying Chaucer. Second, it examined students’ experiences, covering learning practices, resource use, and AI engagement. Third, it explored lecturers’ perspectives on teaching, including their teaching practices, resource use, and experimentation with ChatGPT. This data informed workshops with Chaucer experts and digital humanities developers to explore the creation of a framework for the use of AI in the study of Chaucer’s poetry. The framework involved using AI systems such as ChatGPT and Amazon Polly to develop a digital scholarly edition of Chaucer, with the aim of prompting critical responses from students regarding the use of AI.

The project sample was purposive, focusing on experts, lecturers and students, as well as developers in digital humanities. The sample comprised:

- 62 experts (academic researchers, lecturers and developers) in the fields of Chaucer Studies and digital humanities.
- Six university lecturers who teach Chaucer Studies (three UK and three US).
- 13 undergraduate and postgraduate students (six US and seven UK).
- Four C21 Project researchers and digital humanities developers.

The order of the methods used were:

1. Semi-structured interviews with experts (n. 46).
2. Peer interviews with students (n. 13).
3. Semi-structured interviews with lecturers (n. 6).
4. Two workshops, each with eight participants comprising experts (n. 16).
5. Development of a digital scholarly edition (creating a database and data admin system to import texts; integrating annotation and editing tools to tie in the data; and using design wireframes to develop the resource).

The analytical approach was based on thematic analysis, which was applied to all datasets obtained through the methods described above. The collected data was transcribed and initially open-coded in NVivo, followed by closed coding. The resulting codes were used to develop working concepts and categories. The relationships between these were then interpreted, analysed, and synthesised to identify themes (see below) that informed the findings and final analysis.

The C21 Project received ethics approval from the University of Sheffield Research Ethics Committee and the University College Cork (UCC) Ethics Committee for all interviews and collaborative activities. All contributors provided informed consent to participate and for their responses to be included in the project’s future research outputs.

## Findings

### Resources for studying Chaucer: expert perspectives

The themes that emerged from the interviews with experts were as follows: 1) resources for learning and teaching are in flux; 2) resources extend beyond book technology to include digital resources; 3) collaboration is central in producing resources for learning and teaching.

***Theme 1.*** There was widespread recognition that established resources for learning and teaching are in flux. Experts noted that the use of printed books is now complemented by digital resources in teaching and learning practices. They acknowledged that AI has the potential to enrich study but emphasised that it must be grounded in scholarly knowledge. Some expressed concern that AI may struggle to distinguish between “literary” and “non-literary” writing and may be unable to interpret the form and content of Chaucer’s poetry (Expert 21). As a result, they stressed that students must be taught about representation and editing, in addition to understanding the form and content of Chaucer’s work. This would enable students to critically assess the outputs produced by AI, particularly Generative AI.

Following this point, experts suggested that digital scholarly editions could support the use of Generative AI by providing “a critical representation of a primary source text” (Expert 13), which would serve as an authoritative resource for students and researchers. They also observed that such editions improve accessibility. As one expert noted, they increase access by “making the data … available. … one good example is the Canterbury Tales project” (Expert 33). Some editions also include access to spoken Middle English via apps, for example, “the CantApp where… an actor reads aloud the text” (Expert 33). Digital scholarly editions were therefore seen as important for AI integration, as they offer scholarly knowledge and enhance access to both literary content and spoken Middle English.

***Theme 2.*** The broadening of resources to include oral and digital materials means that academic study has “gone outside of the confines of book technology and … [is] operating in this very fuzzy space where the shape and the form” of resources are changing (Expert 10). This shift raised questions about whether digital scholarly editions, when combined with AI, could “have different functions” (Expert 9). One participant highlighted that existing technologies such as TEI, Omeka, Scalar, and TEI Lite provide “a framework by which people can record, through ‘markup’, their understandings and interpretations of one or more texts and make those accessible to users” (Expert 19). These technologies enable both lecturers and students to contribute to the development of knowledge. Experts also considered whether AI tools could support knowledge creation, although they acknowledged this remains uncertain.

***Theme 3.*** Experts asserted that collaboration is central to developing tools and resources. As Expert 45 stated, “it’s about the people that we work with… in the process of making.” In considering how to develop AI use, experts referenced existing collaborative practices, such as “collaborative review” (Expert 8). These reviews take various forms. For example, in the George Thomason Newsbooks resource, students and lecturers collaborate by adding navigational information, dating protocols, and contextual details (Wessels et al., 2015).^1^ Other examples of participation involved the use of tools like WebGL and citizen science platforms such as *Page*. These tools were seen as part of active learning practices. Experts noted that learning resources are often developed in collaboration with teaching staff and with various forms of student input. They felt that this collaborative approach led to tools that are centred on learning and teaching needs, making it essential to adopt the same approach in developing AI-enabled tools.

In further discussing active learning, experts noted several advantages of digital resources in studying Chaucer. One expert explained that “the digital [has] the capacity to capture the … dynamic side of the oral tradition with the more stable component of the tradition of writing, and … the infrastructure for that is ever evolving and has to be developed” (Expert 17). However, experts felt that little is currently known about how AI applications, such as ChatGPT and Amazon Polly, could be integrated into existing digital resources. To explore this potential, they argued for the importance of understanding student and lecturer perspectives through collaborative research with digital humanities developers. This would help ensure that the needs of learning and teaching are addressed in shaping AI use and would help identify appropriate AI applications and the supporting resources required.

### Learning about Chaucer and using AI: student perspectives

The themes that emerged from interviews with students in Chaucer Studies about learning were: 1) the challenges of understanding Chaucer’s texts and their meaning; 2) their active learning practices; 3) how they were exploring the use of AI.

***Theme 1***. Students reported that studying Chaucer was challenging. They found it difficult to: 1) understand Middle English; 2) engage with content, rhyme and metre of Chaucer’s poetry; 3) understand the social, religious and cultural context of the medieval period; 4) and assess Chaucer’s relevance to contemporary society.

Students drew on lectures, seminars, print-based texts, and digital resources to develop their understanding and knowledge. Grammar lessons were considered important for “getting at the meaning of the text” (UK 3), as well as for understanding how rhyme and metre function in Chaucer’s poetry. Students also applied skills learned in English Language modules to study Middle English grammar. To build confidence, they relied on prior learning experiences. For instance, one student (US 1) felt confident about translating Middle English because he had previously learnt to another language, in his case Latin. Students with backgrounds in Drama Studies also felt more comfortable speaking Middle English than those without such backgrounds.

Students recognised the need to move beyond basic comprehension toward understanding the deeper meaning of Chaucer’s texts (UK 2). They felt supported by lectures and seminars in developing their understanding and beginning to interpret the texts. Many drew on insights from other modules. One UK student, for example, used knowledge from a Religious Studies module to better understand Chaucer’s playfulness with pilgrimage customs, noting it revealed “such a flippant, funny story written from multiple perspectives and in rhyme” (UK 4). The interviews showed that students actively integrated insights from various academic experiences into their study of Chaucer.

***Theme 2.*** Students actively engaged with the meaning and richness of Chaucer’s work. One remarked that Chaucer “introduces you to all of these people from different walks of life that seem very foreign to the present, but then as you get to understand them better, actually they don’t seem that different from lots of people who you know today” (US 2). A UK student highlighted the relevance of Chaucer’s work to contemporary life, saying it “brings to light that human connection … between people of different ages and eras,” and that “to bring that relation back, it’s quite nice” (UK 6). Students noted that engaging with Chaucer required imagining “different people from different life experiences in the same roles as these characters” (UK 5). One student referenced *The Refugee Tales* (Herd & Pincus, 2019) as a modern parallel (UK 6).

Their learning practices included lectures, seminars, printed texts, digital texts, and oral resources. Students found that using read-aloud sessions and diverse formats fostered a sense of community in the classroom (US 3). A UK student noted that engaging with varied resources created “a sense that you’re a lot more involved” (UK 4). Active learning clearly underpinned their study practices.

***Theme 3.*** Active learning extended to students’ decisions about using AI, how, when, and for what purposes. All students interviewed used digital resources in their learning and made suggestions for further development, such as integrating spoken resources into a digital scholarly edition. They were experimenting with Generative AI using ChatGPT and critically reflecting on its potential. Students questioned its reliability and accuracy, conducting exercises that compared their own work with AI-generated outputs.

Key questions they raised included: How reliable is ChatGPT? Is it representative? To investigate, they explored the sources on which ChatGPT’s large language model is trained, how its algorithms function, and whether bias is embedded. One student concluded: “It wasn’t there yet” (US 3) in terms of academic rigour. Another (UK 3) acknowledged that “not every piece of research is 100% accurate in its translation,” but believed improvements could be made. Students generally agreed that Generative AI could improve if fed the “right material,” with one noting: “Then maybe ChatGPT will actually learn and give us a decent translation” (UK 5).

US students tested ChatGPT by writing short essays on scholarly articles they had read, then asking ChatGPT to do the same. They compared the results and found that while ChatGPT’s output lacked depth and missed recent scholarship, it occasionally raised themes they hadn’t considered, highlighting gaps in their thinking. While the content was basic, the exercise helped them “actually get into Chaucer” (US 1).

One student reflected on a professor’s curiosity about AI’s impact, noting: “He’s really curious about the implications of AI and how that’s going to change how we are able to convey information with one another, how we process information” (US 2). Another student observed that professors were not necessarily prohibiting AI-assisted writing but using it experimentally to “help us think through what we are writing and to see AI’s limitations” (US 3). Students understood that while AI tools like ChatGPT could aid learning, they were not substitutes for student analysis, research, or critical thinking.

They agreed with their lecturers that ChatGPT often produced superficial content. For this reason, they believed it could not, and should not, replace their own intellectual efforts. Rather, they saw AI as a supplementary tool. One student even proposed integrating AI into a digital scholarly edition: “If you keep seeing those assistants popping up on every website, maybe [there could be] an AI assistant for Chaucer sitting there in the corner, maybe incorporating the *Middle English Dictionary* into ChatGPT rather than just opening a glossary” (UK 3).

These reflections demonstrate how students, through active learning, build their knowledge. They use resources thoughtfully and critically, gaining the skills to assess both the limitations and benefits of tools like ChatGPT.

### Teaching Chaucer and the use of AI: lecturer perspectives

The interviews with lecturers developed three key themes about teaching Chaucer: 1) active learning underpins teaching Chaucer; 2) a three-phase model of learning; 3) the use of AI in teaching.

***Theme 1***. Lecturers in both the US and UK emphasized the value of active learning. A UK lecturer remarked: “I try not to underestimate how curious and intelligent my students are because often they come up with brilliant questions and really interesting insights, and that really happens when you give the floor to them and when you give them things to work on” (UKL 1). They employed a variety of teaching methods, including lectures, seminars, digital tools, and student-centred activities.

Lecturers highlighted that studying Chaucer requires a diverse skill set. One UK lecturer explained: “You can’t just study these texts as literary documents without having a linguistic dimension to your consideration. Likewise, you only get so far with the language. Pragmatics is a good point about that because if you’re talking about language and the context of a particular text, you need to appreciate that context. And of course, with Chaucer, that context is going to relate to literary as well as cultural issues” (UKL 2).

Thus, studying Chaucer involves textual analysis, linguistic awareness, and historical and cultural understanding. Lecturers draw on a wide range of resources—printed, digital, and oral—to support active learning.

***Theme 2.*** Lecturers described Chaucer learning as progressing through three phases: 1) familiarisation; 2) deepening of understanding; 3) critical engagement.

Phase One involves initial exposure to Chaucer through lectures, seminars, printed texts, digital tools, and AI (e.g., ChatGPT). Lectures provide foundational knowledge, while seminars promote discussion and comprehension. Students are introduced to digital resources such as the *Norman Blake Editions of The Canterbury Tales*.^2^ Some lecturers encouraged students to use ChatGPT for initial exploration, acknowledging its potential to aid in familiarisation. However, they noted limitations: AI-generated content often lacks depth, accuracy, and the ability to distinguish between literary and non-literary contexts. As such, lecturers agreed ChatGPT is helpful only in this first phase. They also felt that students needed to develop their own study skills rather than relying on AI systems and applications.

In Phase Two, students begin to engage with Chaucer’s work from various disciplinary perspectives: language acquisition, linguistics, textual analysis, and medieval cultural context. This stage involves close reading of primary texts, exposure to audio recordings, and interaction with contemporary academic debates. Students bring in their own experiences and analyses, guided by the lecturers. For lecturers this is a crucial transition stage that seeks to get “them to see the technical forms but also then take that next step to being able to interpret” (UKL 3). Seminar discussions and use of advanced resources deepen students’ analytical capabilities, moving beyond the basic tools used in Phase One.

In Phase Three lecturers seek to develop students’ critical thinking. As one lecturer explained, he “deliberately asks students to think about critical approaches to the text and seeing the critical approaches as just as important as the text itself” (USL 4). Students are encouraged to combine academic literacy with life experiences to analyse Chaucer’s relevance, both in his own time and in contemporary society. At this stage, students may be introduced to more advanced resources, such as the *Piers Plowman Electronic Archive*,^3^ which provides rich contextual information for textual traditions that are also found in Chaucer’s *The Canterbury Tales*. Lecturers pointed out that using these types of resources comes “towards the end of the course” (USL 5). They stated that these types of resources have limitations because they are “designed for real specialists” (USL 6). However, they agreed that students can work with these types of resources in the third phase of learning to develop their knowledge.

***Theme 3.*** The lecturers assessed the use of ChatGPT in relation to other teaching practices, such as lectures, seminars, printed and digital texts, and spoken resources. They argued that AI needs to be embedded in study practices: “all the stuff about ChatGPT really does emphasise how much you need a human to be able to sort through data” (UKL 3). When teaching the translation of Chaucer in the classroom, lecturer USL 1 said it was effective to “use ChatGPT and then give students five or six lines and … ask them to think about why ChatGPT translations are inadequate”.

Another issue raised by the lecturers was the lack of time and audio resources available to support the teaching of spoken Middle English. They noted that AI has not yet developed enough to provide spoken Middle English and expressed a desire for more resources to help students hear the language. They agreed there is a strong need for human and social input in study practices when using ChatGPT.

Recognising that students are turning to ChatGPT, and given its current limitations, lecturers suggested that a digital scholarly edition could provide reliable knowledge about Chaucer, his texts, and spoken Middle English. They felt such a resource was necessary because content generated by the large language models (LLMs) that underpin popular Generative AI systems is not accurate enough for teaching. A digital scholarly edition would offer an authoritative knowledge base for students.

### Collaborating to develop a digital scholarly edition to support the use of AI

The collaboration workshop participants (WPs) addressed the points raised by experts, students, and lecturers. They aimed to design a digital scholarly edition that would support the use of AI in studying Chaucer. Three main themes emerged from the workshops: 1) the triangular relationship of learning; 2) the role of experts in developing a digital scholarly edition; 3) the value of developer experience in developing such a resource.

***Theme 1.*** The WPs identified that learning occurs within a “triangular relationship, text-scholar-reader, reinforcing each other” (WP 6). Participants agreed that “the digital emphasises that relationship” (WP 4). There was strong recognition that learning is active, explored through discussions of other learning contexts. One fruitful comparison was the work that museums do. Museums, participants noted, have “learnt different levels of engagement for different user types” (WP 5). They can do this because they offer both physical and digital content and tools, giving learners access to verified information through digital means. This example led to the development of Theme 2.

***Theme 2.*** The WPs agreed that digital technologies play an important role in active learning. They raised questions about how technology might better facilitate students’ understanding. This was discussed in relation to ensuring that design of digital tools and resources supports students and lecturers in active learning (WP 2, WP 4). Regarding the use of AI such as ChatGPT, experts argued that it was not designed with learning and teaching in mind, as it fails to generate content of the required standard, especially in terms of textual meaning (WP 7). Furthermore, it lacks text analysis tools for commenting on and editing texts.

The limitations of ChatGPT could be addressed through the use of digital scholarly editions. These editions offer systematic approaches to transcription across digital scholarly editions and other digitised primary sources, supporting comparability (WP 8). They also involve human editors, who play a vital role in shaping a scholarly edition to support the use of AI. Editors have “a responsibility to be intellectually honest in their representation or re-presentation of that text … in a way that’s consistent and applies certain principles … in a transparent way” (WP 7). Participants suggested that editing could act as a shared principle in guiding the use of AI and digital scholarly editions.

The WPs concluded that digital scholarly editions are particularly important when students use AI in Chaucer Studies. As the content is verified by experts, these editions provide reliable and in-depth resources for students.

The workshops proposed that digital scholarly editions should be part of a broader framework for integrating AI into the study of Chaucer. Such editions can support student learning by providing a validated knowledge base.

***Theme 3.*** Developers considered how the design of a digital scholarly edition should relate to the use of AI in teaching and learning in Chaucer Studies.

To test the claims made by students and lecturers, developers (D1 and D2) used ChatGPT to translate Middle English used in Chaucer’s poetry into Modern English. They used two prompts: one for a literal translation of the text and another that included rhyme and metre. ChatGPT was found to be helpful but still required human editorial review and revision, as approximately 20% of its output was inaccurate. The developers also tested Amazon Polly, a neural text-to-speech service, to produce spoken Middle English. However, it failed to generate accurate pronunciation, often misaligning Middle and Modern English sounds.

These findings validated the lecturers’ earlier points: the human element is essential in the study of Chaucer. While ChatGPT can assist with translation, human experts are still needed to ensure accuracy. In the case of spoken Middle English, only a human voice was found to be convincing. The developers enlisted a professor of Chaucer and spoken Middle English to record *The Pardoner’s Prologue & Tale*, resulting in a version that included accurate pronunciation, rhythm, and intonation. Developers and experts concluded that current AI tools like ChatGPT and Amazon Polly lack access to sufficiently large Middle English datasets to produce reliable content.

The resulting digital scholarly edition^4^ is a prototype that includes the text of *The Pardoner’s Prologue & Tale* in its original Middle English and in Modern English translation. It features annotations on words and phrases, topic guides, digitised manuscript images, and a spoken version. Generative AI was used in translation, annotation, and topic guide creation, drawing on external knowledge sources such as the *Middle English Dictionary* and Wikidata. All AI-generated content was reviewed and edited by human experts and the C21 Project research and development team.

This digital scholarly edition thus combines human scholarly work with the exploratory use of ChatGPT and Amazon Polly. It represents an important component of a broader framework for integrating AI into Chaucer Studies in higher education, offering an authoritative resource that supports active learning.

## Discussion

The findings show that the use of AI applications such as ChatGPT and Amazon Polly in Chaucer Studies is still relatively new, and students and lecturers are currently exploring how to use them. Students, lecturers, and experts were engaging with AI through active learning. Active learning lies at the heart of teaching and learning in higher education and underpins the research conducted by the experts. It was being extended beyond conventional learning, teaching, and research practices to include the exploration and consideration of AI.

The exploration of AI use in Chaucer Studies is taking place at a time when resources for academic study are in flux and increasingly extend beyond the printed book. Digital resources and tools are now integral to learning, complementing lectures, seminars, and printed texts. These resources support active learning approaches in Chaucer Studies. The potential of ChatGPT and Amazon Polly was being assessed by students, lecturers, Chaucer experts, and digital humanities developers. Each group identified concerns, particularly regarding the quality and depth of AI-generated content. While ChatGPT was considered a useful tool for helping students become familiar with Chaucer, more scholarly resources, such as digital scholarly editions, were seen as necessary to support critical engagement and advanced study.

Students actively developed their knowledge of Chaucer, and lecturers employed a range of teaching practices and resources to support student learning. These included lectures, seminars, primary texts, and digital resources. Students drew on their experiences of active learning to experiment with ChatGPT, exploring its uses and limitations. This exploration was reflective, with lecturers guiding students in testing ChatGPT, while students also critically assessed it themselves.

Students and lecturers agreed that AI has limitations, a conclusion supported by both experts and digital humanities developers. ChatGPT was found to be helpful but limited to supporting initial student engagement with Chaucer and his texts. Amazon Polly failed to produce accurate spoken Middle English, requiring a professor with expertise in the language to record the audio. All research participants agreed that a digital scholarly edition is essential for studying Chaucer, as it provides an authoritative resource. In the context of AI use, such an edition serves as a trusted knowledge base that students can use to verify and assess AI-generated content.

The collaborative approach of the C21 Project was valuable in developing a framework for the use of AI in learning and teaching in Chaucer Studies. The collaboration among experts, students, lecturers, and developers enabled the creation of a framework aimed at ensuring that AI is used in ways that support academic study. Students contributed insights from their learning experiences, lecturers discussed their teaching practices, and experts provided scholarly knowledge of Chaucer Studies. Drawing on these perspectives, the C21 Project developed a framework based on student and lecturer needs, supported by the expertise of Chaucer scholars. Specifically, the framework helps students learn about AI and assess its strengths and weaknesses. It supports lecturers in guiding students to use AI reflectively through active learning and provides them with an authoritative resource in the form of a digital scholarly edition. The framework also emphasises the importance of collaboration, as developers in the C21 Project worked closely with students, lecturers, and experts to produce the digital scholarly edition in support of AI use in education.

The research found that active learning is a key component in developing and using AI in teaching and learning. It provides the foundation for encouraging students to engage with AI applications in a reflective manner. Lecturers play a crucial role in guiding students to explore and evaluate AI tools such as ChatGPT, encouraging experimentation and critical testing. To address concerns about AI’s limitations, such as its depth, accuracy of translation, and inability to produce quality spoken Middle English, authoritative resources like digital scholarly editions are essential.

The framework developed by the C21 Project consists of three interdependent components:

1. Active learning is the foundation for learning to use AI.
2. The use of AI must be guided by lecturers to foster reflective and informed engagement.
3. AI use must be supported by authoritative resources, such as digital scholarly editions.

The research found that the development of this framework must be student- and lecturer-centred, while also involving collaboration with experts and digital humanities developers. Such a framework can help mitigate the potential drawbacks of AI applications and support higher education stakeholders in making informed decisions about the integration of AI into learning and teaching (Chan & Colloton, 2024). Its focus on student and lecturer perspectives within Chaucer Studies aligns with the human-centred vision advocated by UNESCO.

## Conclusion

The paper aims to contribute knowledge about developing frameworks for the use of AI in learning and teaching in HE, with a particular focus on a framework for Chaucer Studies. In doing so, it responds to UNESCO’s call to develop frameworks that support the responsible use of AI in education. The research found that active learning plays a crucial role in integrating AI into Chaucer Studies. Active learning enables students to engage with and critically assess the use of AI in their study practices. It helps students determine when and how to use AI as a learning tool (C21 Project Objective 1).

The research identified limitations in existing AI technologies in the study of Chaucer: ChatGPT was unable to generate accurate translations of Middle English, and Amazon Polly failed to produce intelligible spoken Middle English. These shortcomings led Chaucer experts and lecturers to argue that the effective use of AI in learning and teaching requires the support of a digital scholarly edition, including a human-recorded oral reading of Chaucer’s texts (C21 Project Objective 2). Such editions are essential because they provide verified resources against which AI-generated content can be assessed.

The C21 Project demonstrated that in Chaucer Studies a framework-based approach is useful for guiding the integration of AI into learning and teaching. Frameworks can bridge day-to-day educational practices with high-level principles such as those advocated by UNESCO. This means they enable students to explore AI in their learning while offering structured guidance to students and lecturers on when and how to use AI responsibly. Frameworks also help identify the types of support and resources needed when AI is incorporated into study practices.

A collaborative, human-centred approach is essential for developing such frameworks, as it incorporates the diverse perspectives necessary for the responsible and effective use of AI in education. This approach supports both active learning and informed engagement with AI technologies.

However, the study is limited in scope. It is based solely on the C21 Project, which developed a framework specifically for Chaucer Studies. The research considered only one academic discipline, two AI technologies (ChatGPT and Amazon Polly), and one case study involving two universities, one in the UK and one in the US. Further research is needed to explore how to develop similar frameworks in other disciplines, using different types of AI, and across broader HE contexts. In relation to Chaucer Studies, more work is also needed to improve the accuracy of ChatGPT’s Middle English translations and the quality of spoken Middle English generated by Amazon Polly.

Based on the findings of the C21 Project, this paper offers the following recommendations for guiding the use of AI in Chaucer Studies:

- Recognise that active learning is essential in shaping how AI can be meaningfully integrated into academic study.
- Adopt a framework-based approach to support the effective use of AI in learning and teaching practices.
- Use collaborative methods to develop resources, such as digital scholarly editions, that (1) enable the verification of AI-generated content, and (2) create AI tools based on student and lecturer perspectives.
- Develop subject-specific datasets to enhance AI’s ability to generate accurate and contextually relevant content.

## Data Availability

The datasets used and analysed during the current study are available from the corresponding author on reasonable request.

## Footnotes

1 https://www.dhi.ac.uk/newsbooks/nbcontext?about=resource.

2
https://www.chaucermss.org.

3 https://piers.chass.ncsu.edu.

4 https://www.dhi.ac.uk/ai-chaucer.
